# Serological prevalence of the Schmallenberg virus in domestic and wild hosts worldwide: a systematic review and meta-analysis

**DOI:** 10.3389/fvets.2024.1371495

**Published:** 2024-03-28

**Authors:** Melkie Dagnaw, Atsede Solomon, Binyam Dagnew

**Affiliations:** ^1^Department of Veterinary Clinical Medicine, College of Veterinary Medicine and Animal Science, University of Gondar, Gondar, Ethiopia; ^2^Department of Veterinary Pharmacy, College of Veterinary Medicine and Animal Science, University of Gondar, Gondar, Ethiopia; ^3^Department of Microbiology, College of Medicine and Health Science, University of Gondar, Gondar, Ethiopia

**Keywords:** domestic ruminants, meta-analysis, pooled prevalence, Schmallenberg virus, wild ruminants

## Abstract

Schmallenberg virus (SBV) is an arthropod-borne virus that emerged recently in northwestern Europe in 2011 that affects domestic and wild ruminants and induces abortion, stillbirth, and newborns with congenital anomalies. Since its discovery, SBV has spread very rapidly to too many countries in the world. The overall serological investigation of SBV is needed to improve modeling predictions and assess the overall impact on ruminant animals, which helps to design interventions for control and prevention strategies. Thus, this study aimed to estimate the overall serological assay of SBV in both domestic and wild ruminants around the world. This systematic review was conducted as per the Preferred Reporting Items for Systematic Reviews and Meta-Analyses (PRISMA) guidelines. International databases were employed To search for relevant articles. The pooled prevalence with a 95% confidence interval was calculated with a random effects model. The Cochran’s Q test, τ^2^, and I^2^ were used to assess the sources of heterogeneity. In the current meta-analysis, a total of 41 articles were included. The overall pooled proportion of SBV in domestic and wild ruminants was 49 and 26%, respectively. Substantial heterogeneity was observed in studies on domestic ruminants (*I*^2^ = 99.7%; *p* < 0.01) and studies on wild ruminants (*I*^2^ = 97.9%; *p* < 0.01). The pooled prevalence of SBV was significantly associated with publication time, detection techniques, and species of animals. According to the subgroup analysis, the highest pooled prevalence of SBV was reported in cattle (59%), followed by sheep (37%) and goat (18%). In addition to the subgroup analysis based on publication year, the pooled prevalence of SBV infection has become endemic since 2013 (49%) among domestic animals in the world. Of the diagnostic tests used, the highest anti-SBV antibodies (66%) were detected by a virus neutralization test. In this meta-analysis, the major wild animals that were infected by SBV were red deer, roe deer, fallow deer, mouflon, and wild boar. The highest sub-pooled prevalence of SBV was found in roe deer (46%), followed by fallow deer (30%), red deer (27%), mouflon (22%), and wild boar (11%). In general, the prevalence of SBV was high in cattle among domestic ruminants and in roe deer among wild animals. According to the current information provided by this meta-analysis, evidence-based risk management measures should be established to restrict SBV spread in both domestic and wild ruminants.

## Introduction

Schmallenberg virus was detected for the first time in November 2011 in plasma samples collected from cows displaying fever and diarrhea and farmed near the town of Schmallenberg, Germany (1); hence, it is named Schmallenberg virus. The first acute infections associated with SBV were reported in August 2011, while the first malformations in stillborn animals caused by this virus were detected in The Netherlands in December 2011 (2). Since then, nine countries have reported congenital malformations and stillbirths associated with the presence of SBV as of May 2012. The appearance of SBV was first noted in Belgium toward the end of December 2011, followed by the United Kingdom on 22 January 2012. France detected its first SBV case on 25 January 2012, after the virus genome was discovered by RT-qPCR in brain samples from misshapen lambs born on farms situated in the territorial divisions of “Moselle” and “Meurthe-et-Moselle” in northeastern France (3). It infects several domestic and wild animal species, including cattle, sheep, and goats (4) in alpaca (5) red deer, roe deer (6), fallow deer, moose, bison (7), wild boar (8), and several zoo animals (9). Spain reported the first outbreaks of Schmallenberg disease (SBD) in sheep in 2012 (10).

The diagnosis of SBV infection in living adult animals is difficult for veterinarians.

The infection caused by SBV in adult ruminants is primarily asymptomatic or displays symptoms such as febrile syndrome, especially in cattle. These observable clinical manifestations may also be observed in other viral infections, such as bovine herpes virus type 1, bluetongue virus, and foot-and-mouth disease virus. The direct diagnosis of SBV infection can be realized by performing real-time quantitative polymerase chain reaction (RT-qPCR) developed by Friedrich-Loeffler institute in 2011 on the L segment of the SBV genome (11). A protocol targeting the S segment was later developed and showed higher sensitivity. Besides RT-qPCR, the detection of anti-SBV antibodies present in the serum of infected animals can be performed by the indirect method of SBV infection diagnosis. The virus neutralization test (VNT) and enzyme-linked immunosorbent assay (ELISA) have been developed as tools for serological diagnosis.

Schmallenberg virus infection is also classified as one of the emerging infectious diseases of ruminants, with the transmission mediated by *Culicoides* midges (12). The virus is part of the *Orthobunyavirus* genus, which belongs to the Bunyaviridae family. The virus is closely related to the Akabane, Aino, and Shamonda viruses. A variety of *Culicoides* groups, including *Culicoides obsoletus* complex, *Culicoides chiopterus*, and *Culicoides dewulfi*, were linked to the transmission of SBV in Europe during the virus outbreak in 2011/12 (13). Apparent clinical signs of SBV infection in adult cattle are reported to be short-lived. These include loss of appetite, hyperthermia, diarrhea, and reduction in milk production. Infection during the certain critical period of pregnancy between days 47 and 162 of gestation (14) causes neonatal malformation affecting the neuro-musculoskeletal systems (1, 15). The syndrome is known as arthrogryposis-hydranencephaly syndrome (AHS) and is characterized by arthrogryposis, severe torticollis, ankylosis, kyphosis, lordosis, scoliosis, brachygnathia inferior, and neurological disorders. Most of the anomalies were observed in cases of abortions and stillbirths, while some calves may be born alive with various pathologies and behavioral abnormalities (16).

In a study by Veldhuis et al. (17), seropositivity to SBV was significantly associated with decreased reproductive performances and repeat breeder. Since the report of Hoffmann et al. (1), serological and molecular evidence of the virus has been available from several European countries, including Belgium, France, Greece, the UK, Italy, Spain, Luxembourg, Denmark, Poland, Sweden, and Switzerland (15, 18), Turkey (19), and China (20). According to the European Food Safety Authority (EFSA), SBV infection had also been confirmed in approximately 9,000 ruminant herds across Europe, and nearly half of them were reported in France.

In a Belgian sheep farm where SBV emerged and spread between mid-September and mid-October 2011, ewes lambing in January 2012 gave birth to 17% (28/163) of stillborn or newborn lambs presenting typical deformities, while ewes lambing in March 2012 gave birth to only 5% (8/150) of aborted fetuses, and in May 2012, no impact on lambing was observed. In Spain, SBV circulation has been found regionally in livestock, with seroprevalence values ranging between 54.4 and 75.6% (10). The high seroprevalence detected in some species (up to 80%) raises the question of whether wild ruminants play a role in the maintenance of SBV in Europe (21).

In Spain, there are seven wild ruminant species, with red deer (*Cervus elaphus*) and roe deer (*Capreolus capreolus*) being the most widely distributed. Fallow deer (*Dama dama*), mouflon (*Ovis aries musimon*), and Barbary sheep (*Ammotragus lervia*) are less abundant species, with locally significant populations, while Southern chamois (*Rupicapra pyrenaica*) and Iberian wild goat (*Capra pyrenaica*) are more frequent in the mountain ecosystems of northern and Mediterranean Spain, respectively (22). Serological investigations of wildlife animals represent a cornerstone of disease surveillance (23), especially when virus shedding is only transient and the time frame is unknown since antibodies remain detectable for longer periods.

Using recently developed ELISA tools, several serological surveys revealed a large exposure of wild-living ruminant species in Europe, particularly in roe deer (*Capreolus capreolus*) and red deer (*Cervus elaphus*), with seroprevalences ranging from 20 to 90% (2, 6, 24). In Africa, the available research evidence on SBV in cattle is few; however, seroprevalence as high as 61% in Tanzania (25), 56.6% in Ethiopia (26), and 100% in Mozambique (27) were reported. In sub-Saharan Africa, research studies have suggested a possible occurrence of the virus in both wild and domesticated livestock (25, 28).

The distribution of the virus has an ecological association, with the occurrence being higher (2.5 times) in midland than highland. The prevalence of the virus was found to be 72.3% in aborted animals, 53.5% in non-aborted animals, and 71.4 in the birth of weak calves in Ethiopia (29). Populations of some wild species have expanded in recent decades, mainly because of ongoing changes in land use and more intensive game management practices (30). These epidemiological scenarios have been shown to increase the risk of disease transmission among sympatric species (31). In recent times, meta-analyses are a powerful statistical tool used to summarize existing evidence, systematize, and inform specific decisions as well as that could be extended to incorporate economic considerations in a decision analysis framework. It is a more precise estimation of the effect size of an event compared with only one study result (32). This investigation was important for scientific evidence for the researchers and baseline survey collectors regarding the dairy herd reproductive infectious diseases. Furthermore, it supports intervention regarding the prevention and control of the virus. Therefore, this systematic review and meta-analysis aimed to provide an overall estimate of the seroprevalence of SBV in both domestic and wild animals around the globe.

## Methods

The literature search was conducted from 12 August to 20 September 2023. A systematic assessment of published articles reporting the overall proportion of SBV was performed based on the PRISMA checklist (33) (Supplementary material 1). The major working protocol was performed in seven key steps: study eligibility criteria, information sources, search strategy, outcome variable, data extraction, study quality assessment, data synthesis, and statistical analysis. A comprehensive search strategy was made to identify all relevant studies. Databases such as PubMed, Google Scholar, Web of Science, snowball searching from retrieved articles, and other manual methods were used for literature searches to select included studies by two authors (MD and AS) independently. The research question was “What is the pooled prevalence of SBV in domestic and wild ruminants in the world?”

The following Mesh terms were used in electronic database search: “Schmallenbergvirus,” “SBV,” “PrevalenceofSchmallenberg virus” “epidemiology of Schmallenberg virus,” “Schmallenberg virus in cattle,” “Schmallenberg virus in sheep,” “Schmallenberg virus in goat,” “Schmallenberg virus in domestic ruminants,” Schmallenberg virus in wild ruminants, “Schmallenberg virus in wild animals,” “Schmallenberg virus in red deer,” “Schmallenberg virus in roe deer,” “Schmallenberg virus in wild goat,” Schmallenberg virus in wild boar,” Schmallenberg virus in bison, “Schmallenberg virus in buffalo,” “Schmallenberg virus in fallow deer,” and “Schmallenberg virus in mouflon.” Even we have searched articles based on the combination of these words with specific continents and countries as the context. Articles were written in English. All identified studies were imported to Medley software to remove duplicates and scientific citations from the references.

## Study eligibility criteria

### Inclusion criteria

The search was performed by three field experts (Microbiology, Veterinary Pharmacy, and Veterinary Clinical Medicine) to avoid authors’ bias. This meta-analysis includes all of the primary descriptive studies that have been published in the English language that document the occurrence of SBV in domestic and wild ruminants. The inclusion criteria included articles with a clear estimation of the prevalence of SBV. Study animals became domestic and wild ruminants around the globe at any year. Samples had to be collected from animals that had not been experimentally infected.

### Exclusion criteria

Studies that do not have clear and detailed estimates of the proportion of SBV in the affected host were also excluded. Review articles, duplicates, abstract only, qualitative studies, or only KAP (knowledge, attitude, and practice) questionnaire-based studies, book chapters, case reports, editorials, newsletters, and forum discussions, among others were excluded. Intervention studies that lacked baseline data on the association between animal exposure and disease were excluded from the meta-analysis.

### Information sources

The literature search was conducted from August to September 2023. Database sources such as PubMed/PubMed, HINARI, Web of Science, Google, and Google Scholar were used. The included studies were reported from continents in any study year.

### Data extraction

The relevant data were extracted independently by two investigators (MD and AS). Quantitative and qualitative data extraction from the included studies was performed and presented in the form of a table in an Excel spreadsheet. The extracted components encompassed the name of the primary author along with the year of publication, year of study, country, and all domestic and wild species of ruminants, the total number of animals (N), the number of seropositive animals (primary outcome interest), diagnostic methods (ELISA, PCR, VNT), data collection techniques, and ethical considerations. Disagreements were resolved by discussion and consultation with a third author.

### Study quality assessment

Quality assessment was performed to verify the methodological quality of this systematic review (MD and BD). The quality assessment of the included studies was assessed by the Appraisal tool for Cross-Sectional Studies (AXIS) quality tool (34). This quality assessment tool includes different items including study design, sample size justification, sample representativeness, target population, the use of validated measures, diagnosis of statistical methods, sample selection, sample frame, discussion of non-response bias, reporting of funding, and conflicts of interest.

### Data synthesis and statistical analysis

A meta-analysis was carried out using the R software using the “meta prop” function of the ‘meta’ package (35) and “metaphor” (36) in R software. The pooled prevalence and 95% confidence intervals were estimated using the random effects model based on the restricted maximum likelihood approach (REML), which computes within- and between-study variabilities. It was used to perform the overall meta-analysis (overall effect size), pooled odds ratio, heterogeneity, and weight of each study. In addition, graphs and tables were used to demonstrate the prevalence. Considering that the outcome variable is binary (i.e., SBV positive or negative and given only for single groups, the only possible parameter to measure effect size was the raw proportion with 95% confidence intervals) (32). In a logistic-normal random-effect regression model, the logit transformation was utilized to estimate the pooled proportions, and a mixed-effect logistic regression model was employed for the subgroup analysis.

### Investigation of heterogeneity

The Cochran’s Q test (reported as the *p*-value), τ^2^ (between-study variance), and inverse variance index (I^2^) were used to assess the sources of heterogeneity, which describes the percentage of observed total variation between studies that is due to heterogeneity rather than chance. As explained by Higgins and Thompson (37), the I^2^ index was estimated to represent low, moderate, and high heterogeneity, if this corresponds to I^2^ values of 25, 50, and 75%, respectively. Heterogeneity was deemed to be statistically significant if the I^2^ value exceeded 50% and the Q test revealed a *p*-value of less than 0.10. The degree of study heterogeneity has been evaluated using a forest plot diagram. The forest plot diagram displayed the weights, magnitude of effects, and 95% confidence intervals for each study.

### Subgroup sets

To determine specific between-study variability, a subgroup analysis of the proportion of the SBV in ruminants was performed based on study year, methods of diagnosis study location, and species of the animals.

### Publication bias assessment

Publication bias is usually evaluated through a funnel plot in which asymmetry can be assessed visually, beggar rank, and the Egger test. Thus, in our case, publication bias was assessed using funnel plot diagrams and Egger’s regression test.

### Sensitivity and influential analysis

Sensitivity analysis of studies was performed to evaluate the effect of each study on the pooled result. The results showed that the studies were the prime determinants of the pooled result.

## Results

### Article search results

**Figure 1:**
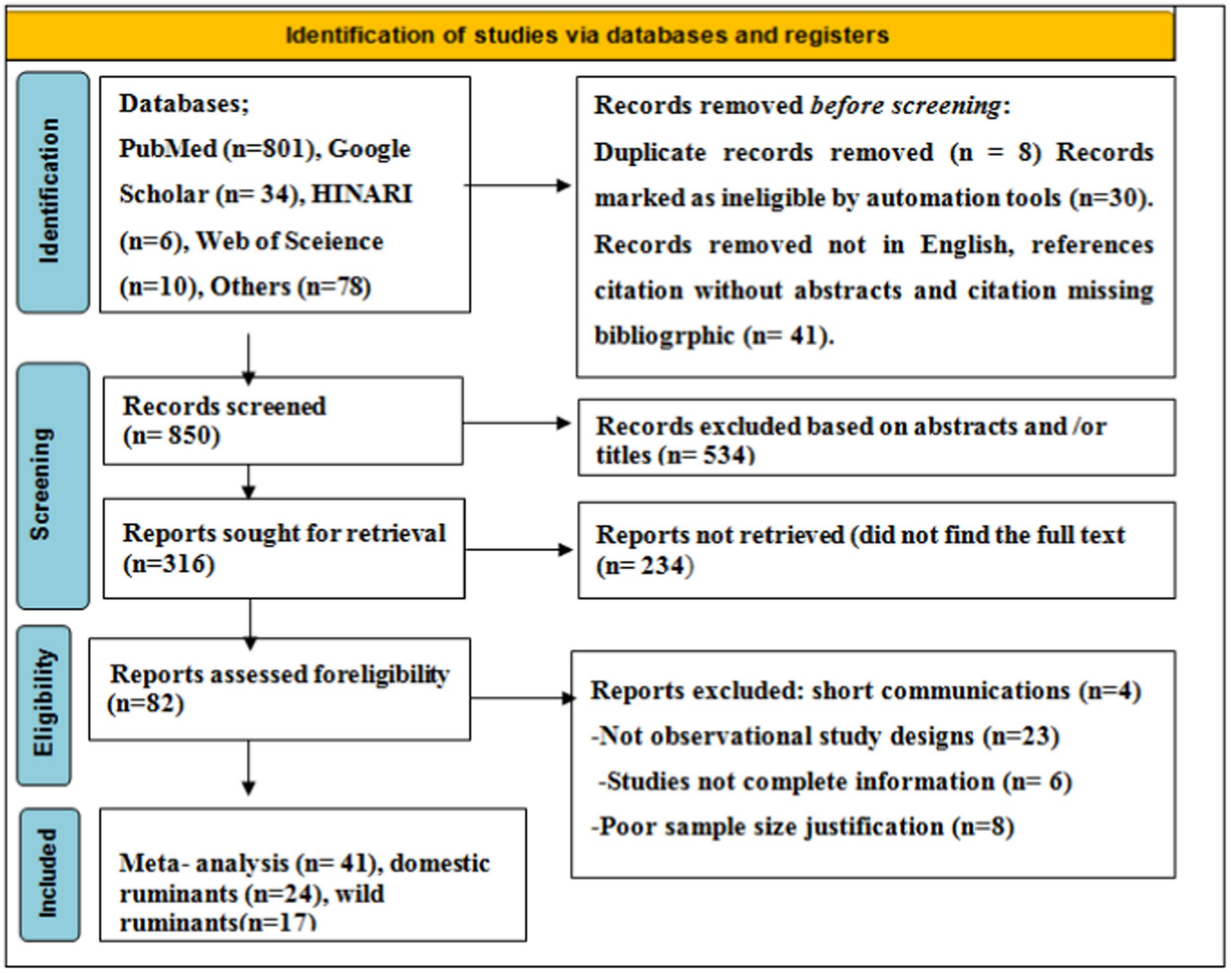
A PRISMA flowchart for searched relevant articles.

As shown in the PRISMA 2020 flowchart (Figure 1), a total of 929 articles in various electronic databases and other methods were searched, from which 8 were excluded after article duplication assessment (*n* = 8), 30 records were marked as ineligible by automation tools (*n* = 30), and 41 records were removed for other reasons (*n* = 41). Among 850 articles, 534 articles were excluded by article title and abstract screening, 316 studies were reports searched for retrieval, and 233 articles were reports not retrieved. A total of 82 (*n* = 82) articles were reports being evaluated for eligibility, and 41 (*n* = 41) of them were excluded for various reasons. Finally, 41 (*n* = 41) studies were included for meta-analysis.

### Overview of included studies for both domestic and wild ruminants (*n* = 41)

The characteristics of the studies about SBV are intricately described in a step-by-step manner. The study animals comprised of cattle which are of all ages, both genders, wild and domestic ruminants in the world. A total of 41 independent articles (domestic, *n* = 24, wild, *n* = 17) were considered for the analysis of all pooled prevalence SBV in wild and domestic ruminants. A meta-analysis was carried out separately for the prevalence of SBV in domestic and wild ruminants.

In domestic ruminants, the included studies for this meta-analysis were conducted and published in different parts of the world between 2011 and 2022. We noticed that the same article was used multiple times due to different species of animals. The study designs were cross-sectional, and the types of samples were serum (blood) and brain tissue. Therefore, it helps to reduce the variability between studies.

The included domestic ruminants were cattle, goats, and sheep with a total number of 909,938 animals and 42,306 SBV positive. In this systematic review, 9,497 cattle (38) served as the maximum sample size, and 19 cattle were used as the minimum sample size (39). Methods of diagnosis tests were ELISA, VNT, and rRT-PCR to examine the presence of the SBV. The majority of these studies were conducted in Europe (Spain, Belgium, France, Greece, etc.) and Africa; four studies were included from Tanzania, Ethiopia, Nigeria, and Mozambique. Moreover, the characteristics of the included studies are depicted in Table 1.

**Table 1:** The characteristics of studies included in the meta-analysis (*n* = 40) for domestic ruminants.

Author	Study year	Country	Animal	Sample	M DX	SD	TAE	Positive
Azkur et al. (19)	2006–2013	Turkey	Sheep	Serum	ELISA	RS	816	325
Barrett et al. (38)	2012–2013	Ireland	Cattle	Brain tissue	RT-qPCR	CS	49	26
Larska et al. (7)	2010–2013	Poland	Goat	Serum	ELISA	CS	121	4
Larska et al. (7)	2010–2013	Poland	Cattle	Serum	ELISA	CS	355	134
Larska et al. (7)	2010–2013	Poland	Sheep	Serum	ELISA	CS	2077	42
Elbers et al. (40)	2011–2012	Netherlands	Cattle	Serum	VNT	CS	1,123	809
Gache et al. (41)	2010–2011	Belgium	Cattle	Serum	ELISA	CS	519	467
Asmare et al. (29)	2012	Ethiopia	Cattle	Serum	ELISA	CC	563	327
Azkur et al. (19)	2006–2013	Turkey	Cattle	Serum	ELISA	RS	1,362	335
Azkur et al. (19)	2006–2013	Turkey	Goat	Serum	ELISA	RS	307	5
Sibhat et al. (26)	2012–2013	Ethiopia	Cattle	Serum	ELISA	CS	1,379	780
Blomstrom et al. (27)	2013	Mozambique	Cattle	Serum	VNT	CS	79	79
Blomstrom et al. (27)	2013	Mozambique	Sheep	Serum	VNT	CS	145	86
Blomstrom et al. (27)	2013	Mozambique	Goat	Serum	VNT	CS	141	112
King et al. (42)	2011–2013	Ireland	Cattle	Serum	VNT	CS	851	396
King et al. (42)	2011–2013	Ireland	Sheep	Serum	VNT	CS	251	164
Mathew et al. (25)	2012–2013	Tanzania	Cattle	serum	ELISA	CS	789	476
Mathew et al. (25)	2012–2013	Tanzania	Cattle	serum	VNT	CS	110	56
Oluwayelu et al. (43)	2012–2014	Nigeria	Cattle	Serum	ELISA	CS	490	446
Oluwayelu et al. (43)	2012–2014	Nigeria	Sheep	Serum	ELISA	CS	165	108
Barrett et al. (38)	2012–2013	Ireland	Sheep	Brain tissue	RT-qPCR	CS	30	11
Barrett et al. (38)	2012–2013	Ireland	Cattle	serum	ELISA	CS	9,497	3,292
Sohier et al. (44)	2015–2016	Belgium	Sheep	Serum	ELISA	CS	409	107
Meroc et al. (45)	2012–2013	Belgium	Cattle	Serum	ELISA	CS	7,130	6,132
Steinrigl et al. (46)	2011–2012	Australia	Cattle	Serum	ELISA	CS	2,113	2070
Veldhuis et al. (17)	2011–2012	Netherlands	Cattle	Serum	ELISA	CS	4,377	3,241
Veldhuis et al. (17)	2011–2012	Netherlands	Sheep	Serum	ELISA	CS	4,379	3,306
Veldhuis et al. (17)	2011–2012	Netherlands	Goat	Serum	ELISA	CS	1,548	782
Gache et al. (41)	2011–2012	France	Cattle	serum	ELISA	CS	1,280	294
Chaintoutis et al. (39)	2012	Greece	Cattle	Serum	ELISA	CS	90	59
Chaintoutis et al. (39)	2012	Greece	Sheep	Serum	ELISA	CS	19	10
Larska et al. (47)	2017–2019	Poland	Goat	Serum	ELISA	CS	365	46
Rasekh et al. (48)	2019	Iran	Cattle	Serum	ELISA	RS	273	34
Ferrara et al. (49)	2020	Italy	Cattle	Serum	ELISA	CS	812	329
Firat Dogan et al. (50)		Turkey	SGC	Serum	ELISA	RS	1,590	463
Firat Dogan et al. (50)		Turkey	SGC	TS,WB	RT-qPCR	CS	1,604	51
Abi-Rizk et al. (51)	2016	France	Sheep	Serum	ELISA	CS	750	122
Wernike et al. (14)	2011–2017	Germany	Cattle	Serum	ELISA	CS	300	17
Kęsik-Maliszewska et al. (52)	2013–2018	Poland	Cattle	Serum	ELISA	RS	21,521	8,046

RS, retrospective; CS, cross-sectional; VNT, virus neutralization test; ELISA, enzyme-linked immunosorbent assay; RT-qPCR, reverse transcriptase polymerase chain reaction; SGC, sheep, goat, cattle; TS, tissue sample; WB, whole blood.

In wild ruminants, the major wild ruminants in this study comprise fallow deer, red deer, roe deer, and mouflon. However, some studies included wild boar, wild goat, bison, buffalo, chamois, and Barbary sheep. Of the included studies (*n* = 24), one study (14) in Germany put only the overall prevalence (fallow deer, red deer, roe deer, mouflon, bison) of the SBV infection in wild animals and did not estimate the prevalence for each wild animal species. There were a total of 11,579 wild animals with 2,477 SBV positive. In this review, 2017 (10) cattle served as the maximum sample size, and five cattle were used as the minimum sample size (53). Methods of diagnosis tests were ELSA, VNT, and rRT-PCR to examine the presence of the SBV. The majority of these studies were conducted in Europe (Spain, Poland, France, Italy, and Germany). Furthermore, the details of the included studies are presented in Table 2.

**Table 2:** The characteristics of studies included in the meta-analysis (*n* = 42) for wild ruminants.

Author	Study year	Setting	Animal spp.	Ethical consideration	Sample	Methdx	S. design	Total examine	Positive	Prevalence
Larska et al. (54)	2010–2013	Poland	Bison	Not stated	Serum	ELISA	CS	86	44	0.512
Larska et al. (54)	2010–2013	Poland	Fallow deer	Not stated	Serum	ELISA	CS	56	0	0.00
Larska et al. (54)	2010–2013	Poland	Red deer	Not stated	Serum	ELISA	CS	370	46	0.124
Larska et al. (54)	2010–2013	Poland	Mouflon	Not stated	Serum	ELISA	CS	20	2	0.1
Azkur et al. (19)	2006–2013	Turkey	Buffalo	Not stated	Serum	ELISA	CS	130	2	0.015
Steinrigl et al. (46)	2012	Australia	Fallow deer	Not stated	Serum	ELISA	CS	10	7	0.7
Steinrigl et al. (46)	2012	Australia	Red deer	Not stated	Serum	ELISA	CS	45	29	0.644
Steinrigl et al. (46)	2012	Australia	Roe deer	Not stated	Serum	ELISA	CS	29	22	0.759
Jiménez-Ruiz et al. (10)	2010–2016	Spain	Fallow deer	Yes	Serum	ELISA	CS	2017	99	0.049
Jiménez-Ruiz et al. (10)	2010–2016	Spain	Red deer	Yes	Serum	bELISA	CS	307	97	0.316
Jiménez-Ruiz et al. (10)	2010–2016	Spain	Mouflon	Yes	Serum	bELISA	CS	118	33	0.280
Jiménez-Ruiz et al. (10)	2010–2016	Spain	Barbary sheep	Yes	Serum	bELISA	CS	36	8	0.222
Jiménez-Ruiz et al. (10)	2010–2016	Spain	Wild goat	Yes	Serum	bELISA	CS	246	49	0.199
Jiménez-Ruiz et al. (10)	2010–2016	Spain	Chamois	Yes	Serum	bELISA	CS	98	10	0.102
Jiménez-Ruiz et al. (10)	2010–2016	Spain	Roe deer	Yes	Serum	bELISA	CS	194	34	0.175
Linden et al. (6)	2010–2011	Belgium	Red deer	Not stated	Serum	IELISA	CS	313	144	0.460
Linden et al. (6)	2010–2012	Belgium	Roe deer	Not stated	Serum	IELISA	CS	211	85	0.403
García-Bocanegra et al. (21)	2006–2015	Spain	Red deer	Yes	Serum	VNT	CS	653	87	0.133
García-Bocanegra et al. (21)	2006–2015	Spain	Fallow deer	Yes	Serum	VNT	CS	197	47	0.239
García-Bocanegra et al. (21)	2006–2015	Spain	Mouflon	Yes	Serum	VNT	CS	140	23	0.164
García-Bocanegra et al. (21)	2006–2015	Spain	Wild boar	Yes	Serum	VNT	CS	109	3	0.028
Barlow et al. (55)	2012	UK	Fallow deer		Serum	ELISA	CS	16	9	0.563
Larska et al. (54)	2013–2014	Poland	Fallow deer	Not stated	Serum	ELISA/VNT	CS	256	81	0.316
Malmsten et al. (56)	2012–2016	Sweden	Fallow deer	Yes	Serum	ELISA/VNT	CS	44	13	0.295
Chiari et al. (24)	2007–2013	Italy	Red deer		Serum	ELISA/VNT	CS	352	21	0.060
Barlow et al. (55)	2010–2012	France	Red deer	Not stated	Serum	ELISA	CS	486	87	0.179
	2010–2013	Poland	Red deer		Serum	ELISA	CS	69	15	0.217
Rossi et al. (57)	2011–2014	France	Red deer	Not stated	Serum	ELISA	CS	983	376	0.383
Barlow et al. (55)	2012	UK	Red deer	Yes	Serum	ELISA	CS	7	5	0.714
Chiari et al. (24)	2012–2013	Italy	Red deer	Yes	Serum	ELISA/VNT	CS	52	21	0.404
Larska et al. (54)	2013–2014	Poland	Red deer	Not stated	Serum	ELISA/VNT	CS	176	44	0.250
Malmsten et al. (56)	2012–2016	Sweden	Red deer	Yes	Serum	ELISA/VNT	CS	22	4	0.182
Mouchantat et al. (8)	2011–2014	Germany	Mouflon	Yes	Serum	ELISA/VNT	CS	44	33	0.750
Laloy et al. (2)	2012–2014	France	Mouflon	Not stated	Serum	ELISA	CS	73	27	0.370
Larska et al. (54)	2013–2014	Poland	Mouflon	Not stated	Serum	ELISA/VNT	CS	71	1	0.014
Desmecht et al. (58)	2013–2014	Belgium	mouflon	Yes	Serum	ELISA/VNT	CS	700	133	0.190
Chiari et al. (24)	2012–2013	Italy	Wild boar	Yes	Serum	ELISA/VNT	CS	107	25	0.234
Mouchantat et al. (8)	2011–2014	Germany	Wild boar	Yes	Serum	ELISA/VNT	CS	1,462	224	0.153
Rossi et al. (57)	2011–2014	France	Roe deer	Not stated	Serum	ELISA	CS	746	371	0.497
Fernández-Aguilar et al. (53)	2013	Spain	Roe deer	Yes	Serum	ELISA/VNT	CS	5	4	0.800
Díaz et al. (59)	2013–2014	Spain	Roe deer	Yes	Serum	ELISA	CS	75	40	0.533
Malmsten et al. (56)	2012–2016	Sweden	Roe deer	Yes	Serum	ELISA/VNT	CS	11	3	0.273
Wernike et al. (14)	2021–2022	Germany	Fallow deer, red deer, roe deer, mouflon, bison	Yes	Serum	VNT	CS	493	69	0.140

ELISA, enzyme-linked immunosorbent assay; VNT, virus neutralization test.

### Meta-analysis, testing heterogeneity, and bias assessment

In the meta-analysis of studies regarding SBV, a total of 41 independent articles were incorporated in both domestic and wild ruminants (domestic, *n* = 24, wild, *n* = 17). However, it should be noted that certain articles were utilized multiple times due to their relevance in similar years but in different animal species. Therefore, a total of 40 dependent and independent articles were included in the case of domestic ruminants, while 42 articles were included in wild ruminants.

### Result of meta-analysis for domestic ruminants

**Figure 2:**
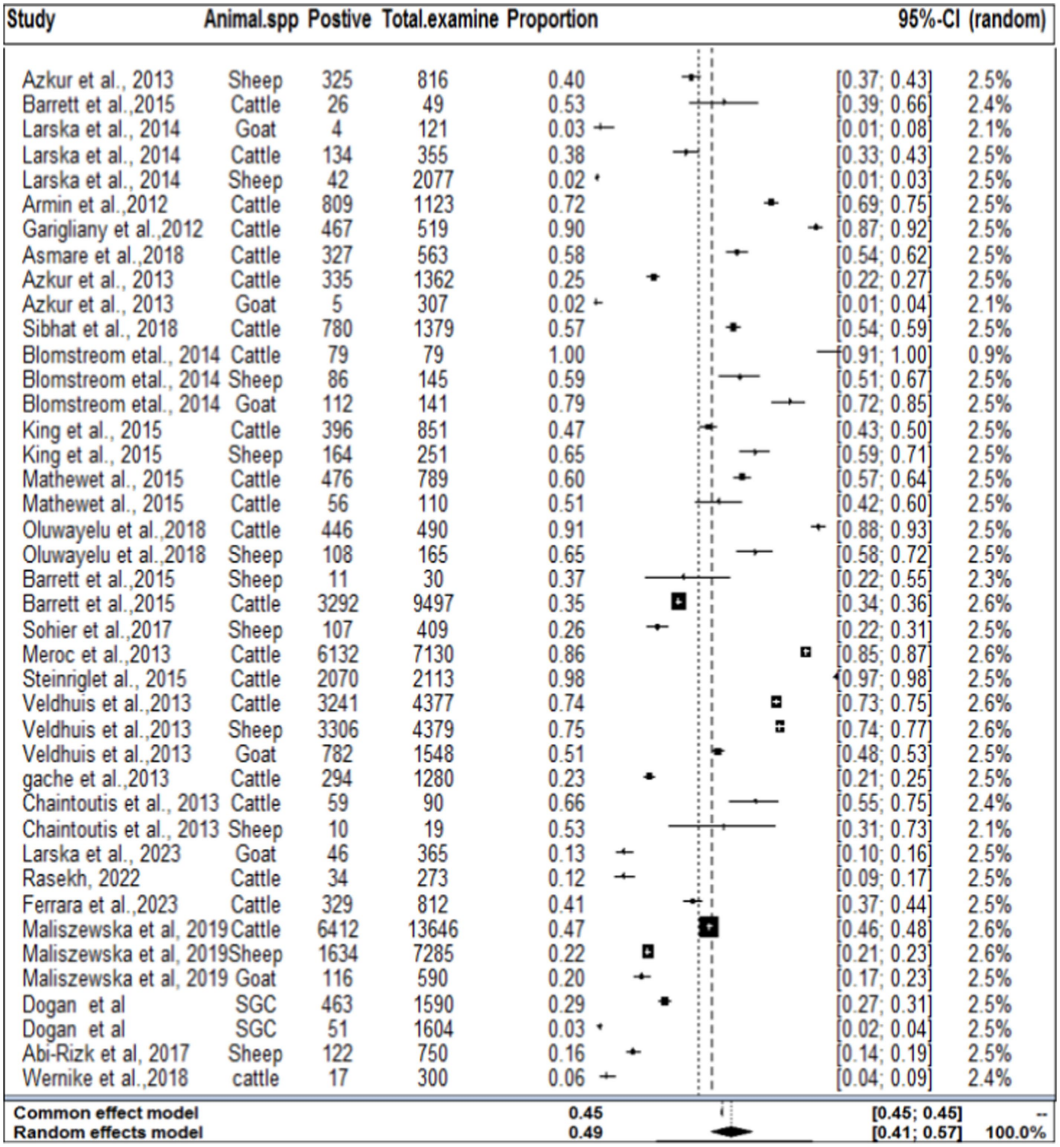
Forest plot plots with random-effects meta-analysis of SBV infection in domestic ruminants.

**Figure 3:**
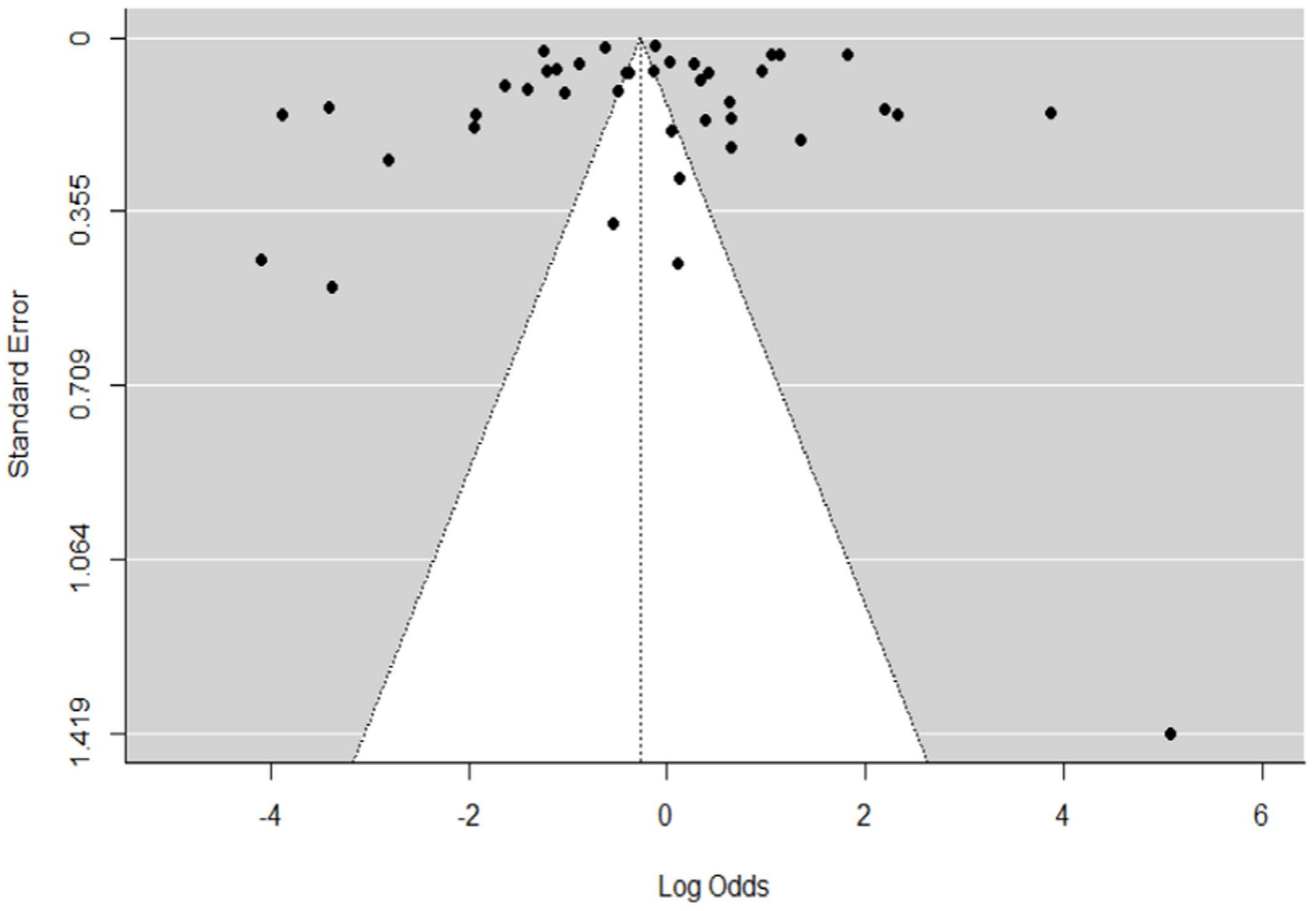
Funnel plot plots the standard error by log odds of the proportion of SBV domestic ruminants.

In the context of domestic ruminants, the included studies exhibited a high level of heterogeneity (*I^2^* = 99.7%: *τ^2^* = 3.0276; *p* < 0.01), and the estimated pooled proportion of SBV among overall domestic ruminants was calculated to be 49% (95% CI: 41–657%; Figure 2). The between-study variability was statistically significant (*Q* = 12262.29, DF = 39, *p* < 0.0001). Similarly, the heterogeneity and outliers of studies were plotted, and the expectation of all studies is not falling within 95% of the range bounded by the two confidence interval lines (Figure 3).

The regression test for funnel plot asymmetry was done using a mixed-effects meta-regression model and standard error as a predictor (Eger’s test, b = −0.2035 (CI: −0.6588, 0.2518), *z* = 0.1008, *p* = 0.9197). The test for funnel plot asymmetry was also performed via rank correlation test for funnel plot asymmetry (Kendall’s tau = −0.0073, *p* = 0.9555).

### Subgroup analysis results

In the current meta-analysis, heterogeneity was explored through a subgroup analysis. Subgroup analyses were conducted based on study year, species of the host or study animals, and methods of diagnosis. All of the subgroup analyses of exploratory outcomes showed considerable heterogeneity (*I*^2^ > 98). Significant statistical heterogeneity in the subgroup analysis reveals a likely interaction among exploratory variables.

**Figure 4:**
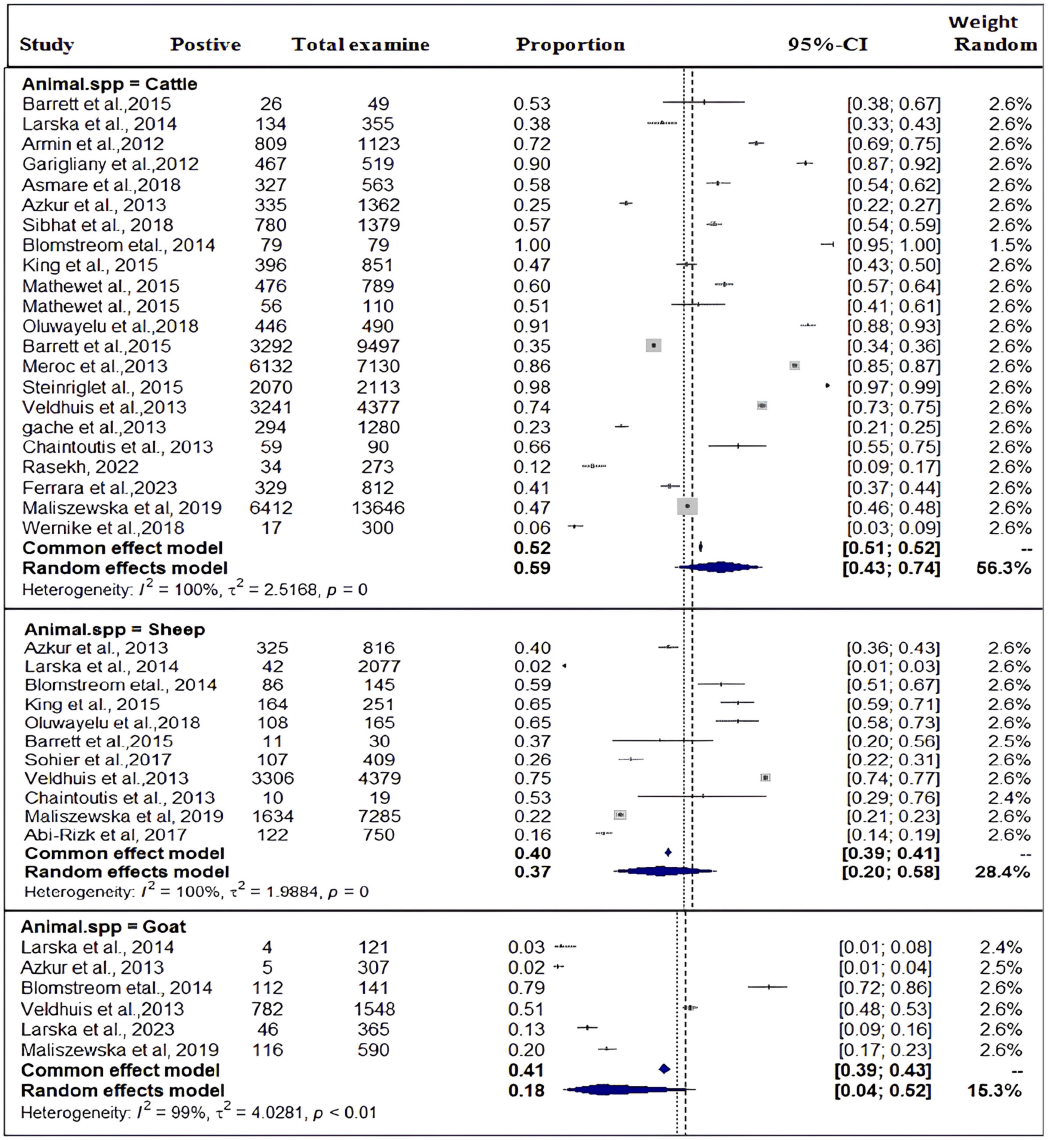
Pooled prevalence of SBV by domestic ruminant species.

Based on the species of ruminant animals, the included studies were categorized into three groups: cattle (*n* = 22), sheep (*n* = 11), and goat (*n* = 6). Significant discrepancies were found in the subgroup analysis of the most SBV prevalence by animal type. As shown in Figure 4, the subgroup analysis revealed that the pooled prevalence of SBV in cattle was 59% (95% CI: 43–74%) and (I^2^ = 100%: τ^2^ = 2.5168=; *p* = 0), followed by sheep at 37% (95% CI: 20–58%) and (I^2^ = 100%: τ^2^ = 1.9884; *p* = 0.0) and goat at 18% (95% CI: 4–52%) and (I^2^ = 99%: τ^2^ = 4.0281; *p* < 0.01).

**Figure 5:**
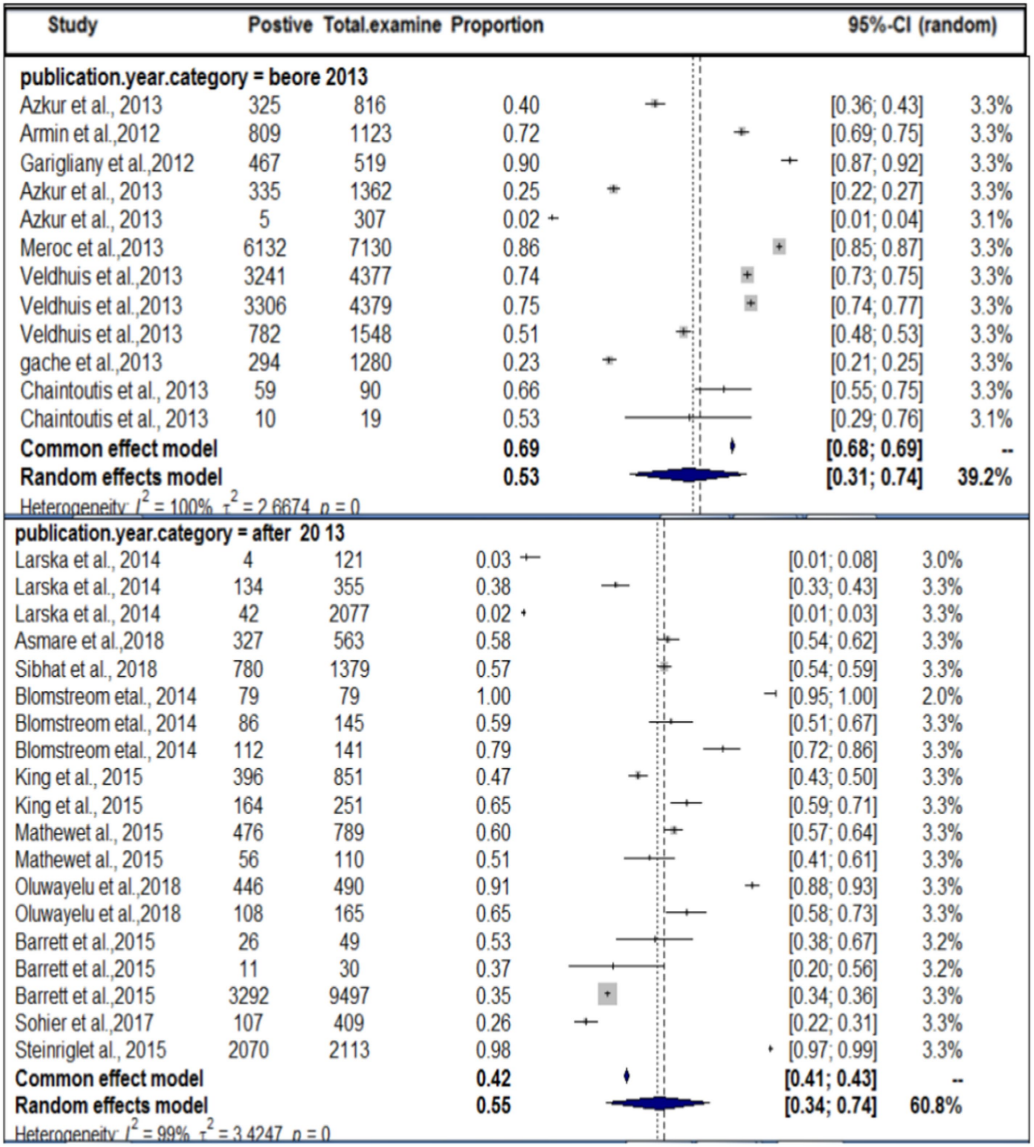
Sub-pooled prevalence of SBV in domestic ruminants by publication year.

In terms of the year of publication, as shown in Figure 5, the subgroup analysis found that the pooled prevalence of SBV virus in domestic ruminants was 27% (95% CI: 25–56%) with 99% degree of heterogeneity (*I^2^*) and (τ^2^ = 3.1833: *p* < 0.01) in the up to or before 2013 group, 49% (95% CI: 25–56%) in the 2013 or later group with 98% degree of heterogeneity (*I^2^*).

**Figure 6:**
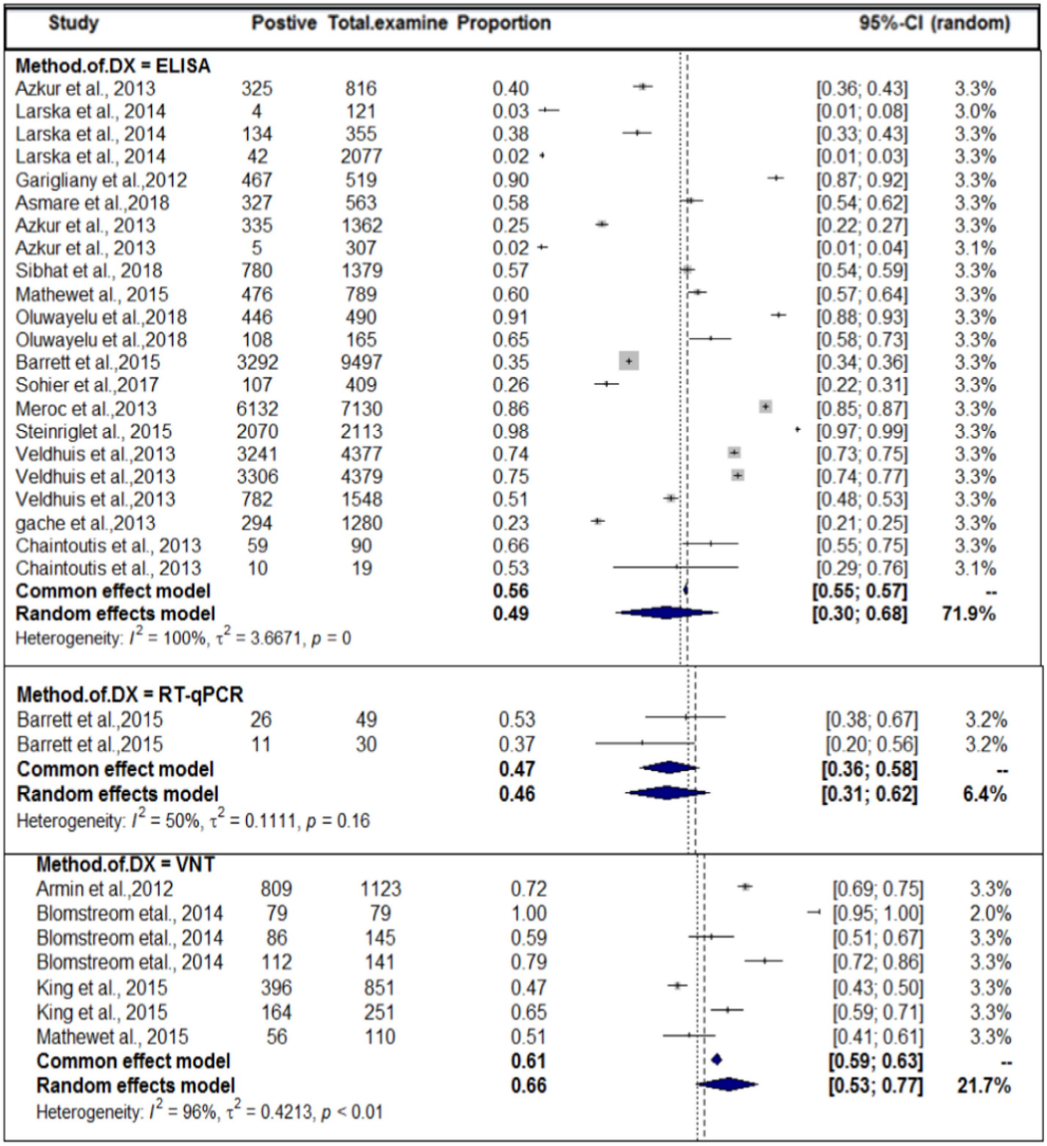
Pooled prevalence of SBV by domestic ruminant species.

In terms of the method of diagnosis, subgroup analysis of the method of diagnosis was carried out. The method of diagnosis was categorized into VNT, ELISA, and RT-qPCR. In this case, we also encountered considerable heterogeneity (I^2^ > 50) in each group. The highest study heterogeneity (I^2^ = 100) was revealed in the diagnosis of SBV by ELISA methods. The sub-pooled prevalence (66%, Figure 6) of SBV was highest in the diagnosis method of VNT, followed by ELISA (49%) and RT-qPCR (46%). The subgroup difference test suggested that there was a statistically significant group effect (Q = 23.49; DF = 2; *p* < 0.0001).

### Result of meta-analysis and subgroup for wild ruminants

**Figure 7:**
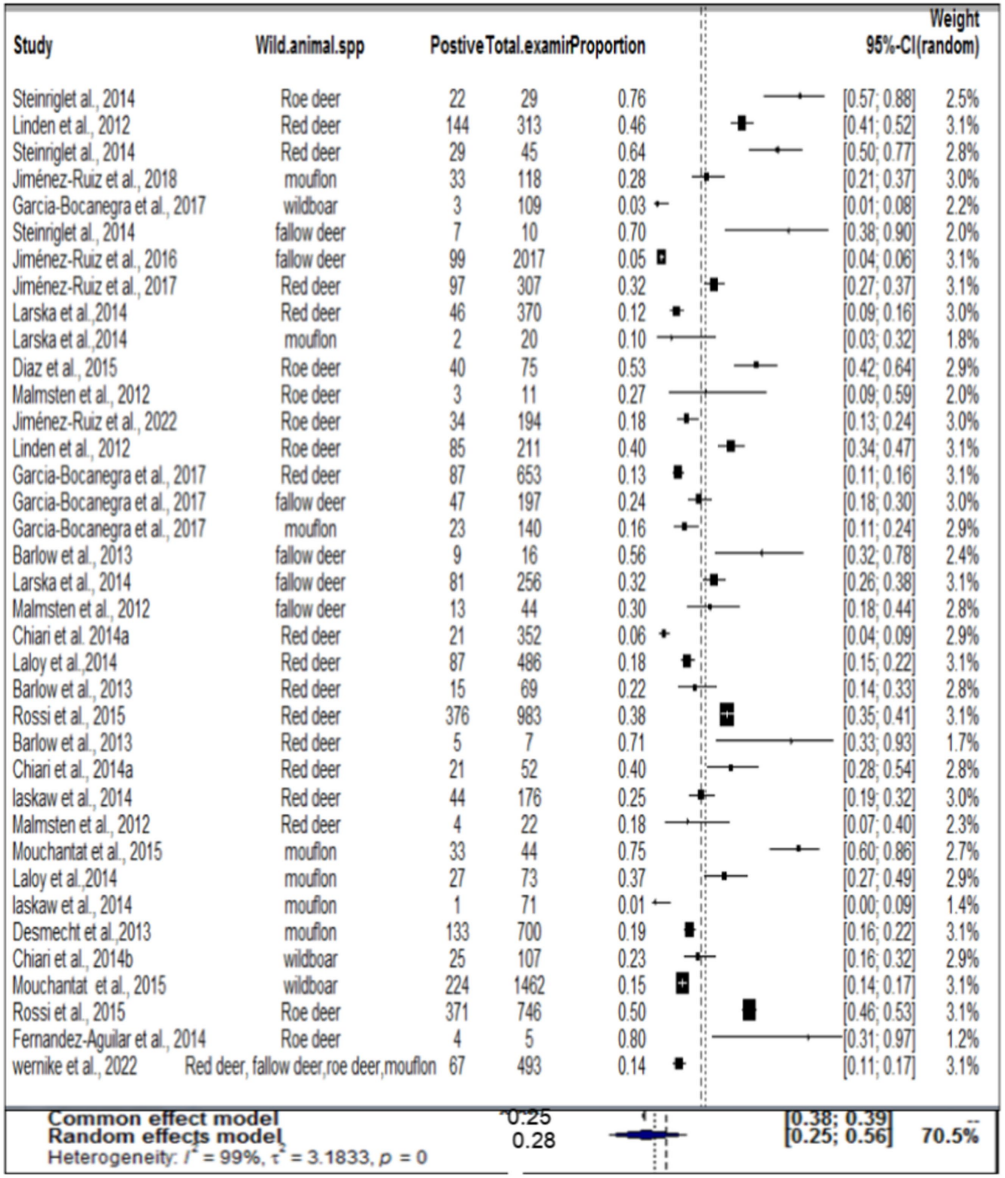
Pooled prevalence of SBV in wild ruminant.

**Figure 8:**
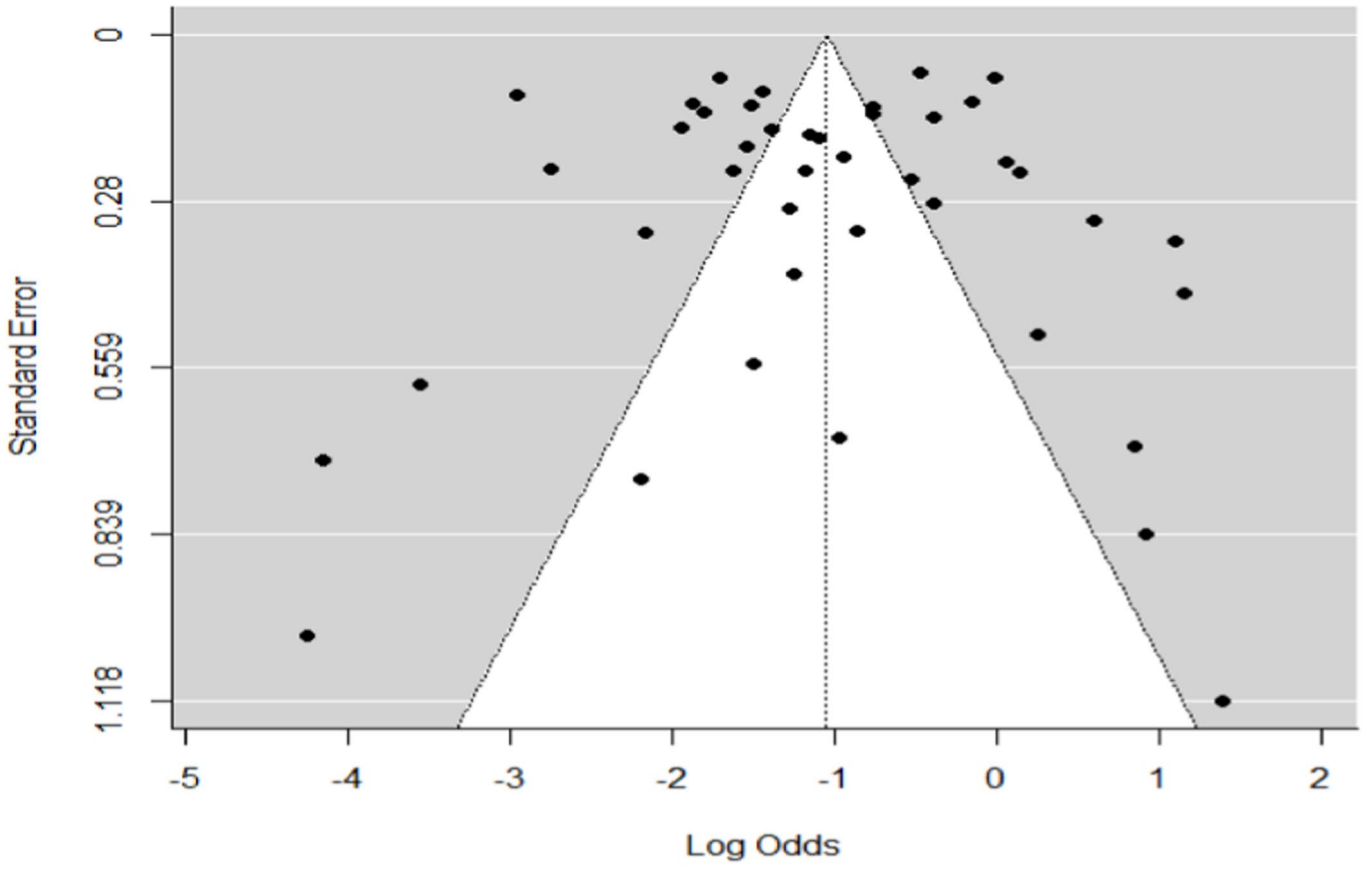
Funnel plot plots the standard error by log odds of the proportion of SBV wild ruminants.

In the case of wild ruminants, the combined prevalence of SBV in wild ruminants was found to be 26%, with a degree of heterogeneity of 97.9% (Figure 7). Funnel plot asymmetry was also conducted for the studies included in wild ruminants, using Egger’s regression test (*b* = −1.0658, CI: −1.5391 to −0.5924, *z* = 0.0768, *p* = 0.9388) and rank correlation test for funnel plot asymmetry (Kendall’s tau = 0.0685, *p* = 0.5319). Therefore, both the funnel plot (Figure 8), Egger’s regression test, and rank correlation test revealed that there was no asymmetrical distribution of studies, indicating that smaller studies were not likely to be overlooked.

Regarding wild ruminants, a subgroup analysis was conducted based on the publication year and type or species of wild animal. The pooled prevalence of SBV infection in roe deer was found to be 33% (95% CI: 24–44%) in the group up to or before 2013, and 26% (95% CI 18–36%) in the group after the publication year of 2013 (Table 3). The estimated pooled prevalence of SBV infection was 46% (95% CI 26–63%) in tested roe deer, 30% (95% CI 13–54%) in fallow deer, 27% (95% CI 18–38%) in red deer, 22% (95% CI 9–45%) in mouflon, and 11% (95% CI 3–32%) in wild boar (Table 3). Among the included studies, a high level of variability was observed in studies focusing on fallow deer, with an I^2^ value of 98 and a *p*-value of <0.01.

**Table 3:** The sub-pooled prevalence of SBV based on wild animal species and publication year.

Moderator	Category	No studies	Total exam	Positive	Pooled prevalence	%CI	Heterogeneity			
							I^2^ (%)	Q (d.f)	tau^2	*p*-value
Species-wise	Roe deer	7	10,490	2,295	46	[26–63%]	92	7.69 (4)	0.749	P < 0.01
	Red deer	13	7,253	1,455	27	[18–38%]	96		0.850	P < 0.01
	Fallow deer	6	4,477	673	30	[13–54%]	98		1.58	P < 0.01
	Mouflon	7	7,783	1,476	22	[9–45%]	92		1.86	P < 0.01
	Wild boar	3	9,234	1,692	11	[3–32%]	87		1.210	P < 0.01
Year-wise	At or before 2013	9	8,170	1,671	33%	[24–44%]	92	1.03 (1)	0.343	P < 0.01
	After 2013	27	10,177	2,151	26%	[18–36%]	98		1.458	P < 0.01
Overall		36	11,579	2,477	26%	[19–33%]	97.95%	1,171 (35)		*p* < 0.001

## Discussion

To the best of our knowledge, this is the first meta-analysis of the global prevalence of SBV infection in wild and domestic ruminants, although many investigations regarding the prevalence of SBV infection in ruminants in different countries have been conducted. The overall serological investigations of SBV are needed to improve modeling predictions and assess the overall impact of SBV in ruminant animals. Wildlife animals may be susceptible to multiple infectious agents of public health or veterinary relevance, thereby potentially forming a reservoir that bears the constant risk of re-introduction into the human or livestock population. A meta-analysis was performed to estimate the pooled prevalence of SBV infection in domestic and wild ruminants in the world. The current meta-analysis includes 34 articles, and it found that the overall pooled prevalence of SBV was 54 and 26% in domestic and wild ruminants, respectively.

In terms of domestic ruminants, according to this finding, the pooled prevalence of SBV (49%) was very high. This finding was comparable to the results (39) in sheep in Greece (53%) (38), in cattle in Ireland (53%) (25), in cattle in Tanzania (51%), and (17) in goat in the Netherlands (50%). The consistency in the results between the three serological surveys, in itself, suggests that there was limited bias in sample selection, equal vaccine cover, and equal exposure to the vector. The result was lower than the results reported by Refs. (27, 45, 46, 60) in cattle in the Netherlands (72%), Mozambique (79.4%), Australia (98%), and Belgium (86%), respectively. In contrast, the estimated pooled result was much higher compared with Refs. (7, 19, 44). in Poland (2%), Turkey (24%), and Belgium (26%), respectively.

However, some of the researchers have found different findings in the same area, and the same time, and even in the same species, for instance, the percentages of seropositive cattle reached 90.8% (8) and 99.76% (19) in Belgium and France up to 79% (7). The seroprevalence reported at the same time in sheep and goats differed significantly. For example, SBV seroprevalence in small ruminants did not exceed 58.7 and 43.8% in Germany. In Greece, the difference between the percentages of seropositive samples from cattle (64.45%) and sheep (5.3%) also was significant. A British study has also shown rather low seropositivity in sheep flocks investigated. No seropositive ewes were found in some flocks despite confirmation of SBV infection in the lambs originating from the same flocks and grazing close to seropositive cattle farms. In the Netherlands, the seroprevalence varied depending on the production type with 98.5, 63.4, 89.5, and 50.8% of seropositive animals among beef cattle, dairy cattle, sheep, and goats, respectively (23). These differences may be related to the availability of vaccination, the season of sample collection, the geographical location farm, the clinical stage of the virus during sampling, farm management systems, and the density of the vector (*culicoides*). Probably, environmental characteristics such as humidity and variation in temperature during the night and day affect the density of the vector, influencing the possible transmission and consequently the prevalence of SBV in both domestic and wild ruminants.

A Scottish SBV model indicates that the introduction of SBV must occur relatively early in the vector season to bring about extensive spread (61). Furthermore, stocking densities, manure in farms, land use, and meteorological conditions are considered to account for the differences in infection pressure (62). Moist soils rich in organic matter such as those created by dumping manure and other farm wastes may well support the breeding of *Culicoides* spp. Agroecological differences between the locations of the farms had a significant effect on the seroprevalence of SBV. Farms located in the midland areas had higher seroprevalence than those located in the highlands. This finding could be associated with favorable environmental conditions created by factors, including warmer average temperatures and moisture that support insect breeding and multiplications in the low-lying areas (63). Virus transmission and spread are possible at temperatures around 15°C with an optimum temperature range between 18°C and 19°C due to vector limitations (64).

In the subgroup analysis, the overall prevalence of SBV in cattle (59%) was highest compared with small ruminants, which is consistent with the result reported by Chaintoutis et al. (39) in cattle in Greece and (25) in cattle in Tanzania. Several studies have shown that cattle are more attractive to biting midges than other ruminant species, which makes them the ideal sentinel species for a midge-borne disease. However, a search of the literature indicates that, to date, there is no direct evidence that such resistance or susceptibility mechanisms of cattle for SBV. The current meta-analysis has revealed that the pooled prevalence of SBV was higher in sheep (37%) compared with goats (18%). It was proposed that this discrepancy in seropositivity between sheep and goats in Belgium and the Netherlands is a result of the animals’ husbandry practices, with sheep being more commonly kept outdoors (17). Furthermore, the timing of the reproductive season, particularly in sheep flocks with early breeding seasons (which coincided with the active season of the *Culicoides* vector), has also been identified as a risk factor for congenital Schmallenberg disease (65).

By study year subgroup analysis, a considerable increase in the seropositivity rate was observed in animals that were tested after 2013 compared with animals tested in or before 2013, indicating a re-circulation of SBV and seroconversion of naive young stock. May also related to the probability of the population of the vector density, if the vector density is increased in a specific area the disease may be transmitted and increase the probability of exposure. The virus entered a virgin population within 13 years, became endemic. This high seroprevalence is also the result of prolonged immunity (demonstrated in several studies) and continuous exposure (in many countries, the vector season is now very prolonged). Climatic conditions, local densities of vectors, the competence of vector species, and the topography have been recognized as factors that can affect the infection rate among species of farm animals. The occurrence of very cold weather, combined with wet and windy climatic conditions, is likely to result in a delay in the resumption of midge activity (66). The interactivity of *Culicoides* (where SBV replicates to transmissible levels within the midge) is dependent on the extrinsic incubation period (EIP), which is believed to vary between 9 and 41 days depending on the microclimatic temperatures found on farms (67). In Germany, it has been reported that midge activity is minimal during cold weather (68), whereas in warmer conditions, SBV has the potential to spread rapidly. This observation may be attributed to temperature-dependent factors, such as the midge biting rate, extrinsic incubation period, and vector mortality rates, all of which are known to influence the transmission of SBV between animals (68). The adult midges from the *Culicoides obsoletus* group possess a striking ability to endure long periods of time without a blood meal. Specifically, they can survive for up to 10 days at 4°C and up to 92 days at temperatures ranging between 17°C and 35°C (69). These findings suggest that infected midges have the potential to persist during the colder months of the year and subsequently infect hosts once temperatures rise to levels that are more conducive for virus transmission. This proposition is further substantiated by the presence of evidence suggesting the transmission of SBV (Schmallenberg virus) in Germany during the winter of 2013 (14).

The second objective was related to the distribution of SBV in wild animals. In this analysis, the pooled prevalence of SBV virus in wild animals was 24%. In terms of wild animal species, the peak seroprevalences observed in roe deer (46%) are similar to those reported in roe deer in Belgium and France (6, 57). High seroprevalence, ranging between 27.3 and 80.0%, was recently also detected in roe deer in different regions of Spain during the 2013–2014 period. In the last few years, serosurveys have revealed widespread exposure to SBV among wild artiodactyl species in different countries. The prevalence of SBV has been observed in roe deer (*Capreolus capreolus*) (ranging from 17.3 to 75.9%) (10, 46), red deer (*Cervus elaphus*) (ranging from 6.0 to 71.4%) (24, 55) in Australia and UK, fallow deer (*Dama dama*) (ranging from 5 to 70%) (10, 46) in Australia and Spain, bison (*Bison bonasus*) (51%) (7) in Poland, chamois (*R. rupicapra*) (10%) (10) and mouflon (*Ovis aries musimon*) (ranging from 10 to 75%) (8, 54) in Poland and Germany, and wild boar (*Sus scrofa*) (ranging from 3 to 23.4%) (21, 24) in Spain and Italy. SBV seropositivity was determined only from samples positive by both bELISA and VNT. Several sera positive by bELISA could not be tested by VNT due to serum cytotoxicity, so seroprevalence may have been slightly underestimated. Similar peak seroprevalences were also reported in domestic species from the Netherlands, Belgium, Germany, Sweden, and Spain (24, 53, 70).

Commercially available ELISA assays are sensitive, specific, and robust, but cross-reactivity with other members of the Simbu serogroup has been reported for the assay used in this study previously Consequently, when interpreting the results of this and similar ELISAs, care must be exercised because a positive result may not indicate infection with SBV but could be due to infection with another Simbu serogroup virus. While VNTs are considered to be the ‘gold standard’ for the assessment of other assays, it is well recognized that even they are prone to cross-reactivity for viruses belonging to the Simbu serogroup. As the ELISA is relatively easy to perform, requires minimal laboratory equipment, and laboratories do not need to have all reference viruses, it will be preferred in many of the regions where multiple Simbu serogroup viruses may be present. There is a need to validate ELISA kits for use in these endemic areas but this will be challenging due to the complex cross-reactivity (71).

This investigation presents compelling evidence of SBV infection in both domestic and wild ruminants worldwide. The findings of the present study appear to suggest an increase in the spread of SBV after the year 2013. Additionally, our research also indicated a high distribution of SBV in both domestic and wild ruminants. Nevertheless, certain aspects lack documented studies, while other countries have recently discovered the presence of the virus, such as Ethiopia, where the SBV was detected around the year 2018. Specifically, the current study revealed a lack of comprehensive information regarding the propagation of SBV in wild animals. In certain regions of the world, the seroprevalence of SBV is significantly high, yet it receives minimal attention, particularly about dairy cattle in African countries. This research also aids in enhancing our comprehension of the range of species susceptible to the virus and evaluating the effectiveness of current SBV serological assays in both wild and domestic ruminants. Therefore, further investigation is required concerning the spread of the virus in wild animals, as well as the implementation of preventive and control measures, such as vaccine production, in countries with high SBV prevalence.

There are several limitations in our meta-analysis and included articles. First, our systematic review only includes published articles, resulting in the potential reported bias. Second, the subgroup analysis was limited to only publication year, geographical location, or detection method as moderators for investigation of the source of heterogeneity between studies, and the included studies did not cover all wild ruminants. Third, the articles we found did not cover all regions of the world, as some countries had no published articles, leading to potential publication bias. The final limitation was that our review was not registered in the PROSPERO database.

## Conclusion

The current meta-analysis demonstrates the significant rate at which the overall prevalence of SBV was higher in domestic ruminants (49%) compared with wild ruminants (26%). Within domestic ruminants, the present study reveals that the overall serological assay was greater in cattle in comparison with small ruminants. Among wild ruminants, the roe deer exhibited the highest seropositivity. This investigation includes a substantial number of European studies, indicating a better comprehension or possibly a high distribution of the intermediate host (vectors). Based on the available evidence regarding its emergence in 2011, we presume that the global coverage of the virus is extensive. Particularly, this virus will exert a significant impact on dairy industries. Therefore, it is imperative to pay close attention to this disease to counteract its rapid dissemination worldwide. Additionally, certain countries should prioritize early diagnosis for both domestic and wild ruminants.

## Data availability statement

The original contributions presented in the study are included in the article/Supplementary material, further inquiries can be directed to the corresponding author.

## Author contributions

MD: Conceptualization, Formal analysis, Methodology, Software, Validation, Writing – original draft, Writing – review & editing. AS: Conceptualization, Methodology, Writing – original draft, Writing – review & editing. BD: Conceptualization, Formal analysis, Software, Writing – review & editing, Data curation, Funding acquisition, Investigation, Methodology, Project administration, Resources, Supervision, Validation, Visualization, Writing – original draft.
